# Physiological and Transcriptional Regulation of Salt Tolerance in *Thinopyrum ponticum* and Screening of Salt-Tolerant Candidate Genes

**DOI:** 10.3390/plants14172771

**Published:** 2025-09-04

**Authors:** Ran Zhang, Rui Zhong, Kuiju Niu, Fang Jia, Yuehan Liu, Xiaoxia Li

**Affiliations:** 1 Institute of Ecological Protection and Restoration, Chinese Academy of Forestry, Beijing 100091, China; zhangran@caf.ac.cn (R.Z.); ruizhong@caf.ac.cn (R.Z.); jiafang@caf.ac.cn (F.J.); 2 College of Pratacultural Science, Gansu Agricultural University, Lanzhou 730070, China; niukj@gsau.edu.cn; 3 Grassland Research and Technology Promotion Department, Xinjiang Uygur Autonomous Region Grassland Station, Urumqi 830000, China; 320210035@xjau.edu.cn

**Keywords:** *Thinopyrum ponticum*, salt stress, transcriptional regulation, oxidative damage

## Abstract

The tall wheatgrass *Thinopyrum ponticum* has excellent saline–alkali tolerance and great potential for restoring saline–alkali land to enhance productivity. This study used the *Thinopyrum ponticum* cv. “Orbit” variety, which is widely planted in saline–alkali pastures, as the material and artificially simulated salt stress using 150 mM NaCl and 150 mM Na_2_SO_4_, respectively. The growth and physiological indexes of the leaves and roots of seedlings were measured after various treatment durations, and the transcriptomes of untreated and Na_2_SO_4_-treated leaves and roots were also analyzed after 24 h of treatment. The results showed that salt stress resulted in significant reductions in leaf relative water content in seedlings and inhibited root elongation growth, with Na_2_SO_4_ treatment producing a greater impact on plant growth than NaCl treatment. Salt stress significantly alters ion transport and distribution in *Thinopyrum ponticum*, characterized by pronounced Na^+^ accumulation and a concomitant decline in K^+^ uptake. Additionally, to adapt to salt stress, roots enhance their ability to absorb and transport essential cations, such as Ca^2+^, Mg^2+^, Fe^3+^, and Cu^2+^. RNA-Seq analysis identified 1682 and 2816 differentially expressed genes (DEGs) in leaves and roots under Na_2_SO_4_ stress, respectively, with 210 common DEGs. Enrichment analyses revealed that DEGs were primarily associated with redox homeostasis, ion balance, and signal transduction. Furthermore, transcription regulation analysis indicated the *Thinopyrum ponticum* can coordinate the activation of NAC/MYB/WRKY transcription factors, SA/ETH hormone signaling, and Ca^2+^ pathways in response to salt stress. In summary, this study systematically reveals for the first time the molecular mechanisms by which *Thinopyrum ponticum* responds to Na_2_SO_4_ stress through coordinated regulation of ion transport, transcription factor networks, and hormone-Ca^2+^ signaling pathways. Based on transcriptomic and protein–protein interaction analyses, nine key candidate genes for saline–alkali tolerance were identified, including *UGT7472*, *JMT*, *T4E14.7*, *CAX5*, *CP1*, *PXG2 NAMT1*, *BON3*, and *APX7*. These findings provide novel genetic resources and a theoretical foundation for breeding salt–alkali-tolerant crops.

## 1. Introduction

Soil salinization leads to the deterioration of ecological environments and the reduction in a land’s comprehensive production capacity, resulting in severe restrictions and limitations in plant growth and the development of agriculture and animal husbandry [1]. More than 800 million hectares of land (c. 6% of the world’s total land area) are impacted by excess salt concentrations [2]. Thus, cultivating salt-tolerant varieties through molecular and plant breeding is an appealing option to improve the utilization of saline soil and restore soil productivity [3].

By employing appropriate cultivation techniques, modest grain yields can be obtained from grain crops planted on mild to moderate saline–alkali land with salt contents ranging from 0.2% to 0.4%. However, grain yields are often low and volatile from moderate to severe saline–alkali land with salt contents greater than 0.4%, though the development of salt-tolerant forage grass has great agricultural potential [4]. Due to its excellent salt tolerance, the tall wheatgrass *Thinopyrum ponticum* is often used as the primary plant for pasture establishment and soil restoration in saline–alkali land in the United States, Canada, and some European countries. It was introduced in China in the 1980s and has long been used as an important genetic resource for wheat genetic improvement [5]. It is a low-maintenance and salt-tolerant forage grass and ecological grass species with great development potential in China. Nevertheless, little is known about the molecular basis for salt tolerance in *Thinopyrum ponticum*.

Salt stress is commonly caused by high concentrations of Na^+^, Cl^−^, or SO_4_^2−^ in soils [6]. High concentrations of Na^+^ in plant cytoplasm can disrupt the normal function of plasma membrane transporters, hinder or destroy the absorption of water and other nutrients such as K^+^, Ca^2+^, and Mg^2+^ by plants, and affect their normal physiological metabolism [7]. Excessive salt ions typically cause damage to plants by causing osmotic stress, ion toxicity, and oxidative stress. Plants relieve or withstand salt stress damage by regulating stomatal apertures, synthesizing osmotic substances, isolating excess ions, or scavenging reactive oxygen species (ROS) [8]. These responses are regulated by complex signal network systems that include signal perception, signal transduction, transcriptional regulation, gene expression, and the effects of resulting products. Ultimately, it restores ion and osmotic balance to maintain enzymatic activity, while also controlling and repairing damage to alleviate the inhibitory effects of salt stress on plant growth [9].

Ca^2+^ is an important second messenger in plant cells. Upon external stimulation, specific receptors produce Ca^2+^ signals in cells, which regulate downstream response genes that enable plants to cope with stress [10]. The salt overly sensitive (SOS) pathway was the first CBL-CIPK pathway identified in plant cells that functions to maintain ion homeostasis and can enhance salt tolerance in plants through multiple downstream pathways [11]. In the SOS pathway, plants sense salt stress through the salt receptor GIPC (Gly-cosyl inositol phosphorylceramides). The calcium channel on the plasma membrane is then opened, which leads to an increase in intracellular Ca^2+^ concentrations, though the calcium channel proteins that are regulated by GIPC and mediate Ca^2+^ transport in this process are still unclear [12]. The SOS signal transduction cascade is activated in the cells via the Ca^2+^ signal, which promotes the discharge of Na^+^ from cells to maintain Na^+^/K^+^ [13]. In addition, a series of CaCAs (Ca^2+^/cation antiporters), including CAX (H^+^/cation exchanger), CCX (cation/Ca^2+^ exchanger), MHX (Mg^2+^/H^+^ exchanger), and NCL (Na^+^/Ca^2+^ antiporters like protein), as well as Ca^2+^-ATPase, calmodulin (CaM), and calmodulin-like proteins (CMLs), play important roles in maintaining calcium homeostasis [14,15].

Salinity-stressed plants have long been known to exhibit dramatically altered levels of many endogenous signaling molecules, including abscisic acid (ABA), ethylene (ETH), salicylic acid (SA), ROS, and others [16,17]. Salinity promotes the production of ethylene in various species by modulating the activity of enzymes regulating ethylene biosynthesis. 1-aminocyclo-propane-1-carboxylic acid (ACC) synthase (ACS) is a key and rate-limiting enzyme in the ethylene synthesis pathway; thus, the regulation of ACS is critical for ethylene biosynthesis and salinity stress [18]. Early studies showed that the expressions of *ACS5* and *ACS7* were significantly elevated under high salt stress [19,20], and a recent study of rice found that salt treatment promoted the biosynthesis of ethylene and resulted in the up-regulation of *ACS1*, *ACS5*, and *ACO1* [21]. However, the *Arabidopsis acs7* mutant showed reduced ethylene production and enhanced salt tolerance during seed germination [22]. A report has indicated that ethylene treatment of rice plants confers salt hypersensitivity [23]. Hence, the dynamic balance of ethylene plays a complex role in the response to salinity stress. Further, SA is considered to be important in maintaining plant growth and development under high salinity and ETH. A recent study suggested that SA can reverse the negative effects of salinity stress and stress-induced ETH production by modulating the expressions of *ACS*, *NHX* (*sodium–hydrogen exchanger*), *SOS1* (*salt overly sensitive 1*), and high-affinity K^+^ transporters (*HKT1* and *HKT2*), improving various growth and physiological parameters and antioxidant enzyme activities in tomatoes [24]. The crosstalk between SA and ETH supports adaptive responses in plants under salinity stress by regulating ROS generation, ions homeostasis, and programmed cell death (PCD) [25].

In recent years, considerable advances have been elucidated regarding the physiological and molecular mechanisms underlying salt tolerance in gramineous plants. Research demonstrates that humic acid confers enhanced resilience to NaCl stress in *Lolium perenne* through the modulation of pyrroline-5-carboxylate synthase gene expression and associated hormonal reprogramming [26]. In *Suaeda salsa*, the coordinated up-regulation of key Na^+^ transporter genes (e.g., *SsHKT1;1*, *SsSOS1*, *SsNHX1*) facilitates sodium sequestration within leaf vacuoles, thus mitigating ionic toxicity [27]. Furthermore, Xiao et al. established a comprehensive evaluation framework for assessing salt tolerance in *Thinopyrum ponticum* by simulating saline environments with NaCl and Na_2_SO_4_, integrating multi-stage physiological indices including seed germination, seedling emergence, and early growth dynamics [28]. However, salt stress research is still overwhelmingly focused on NaCl, whereas Na_2_SO_4_-induced stress has received scant attention to date. Unlike Na^+^, K^+^, and Cl^−^, SO_4_^2−^ is a distinct ionic species, and Na_2_SO_4_ exerts greater phytotoxicity than NaCl [29,30]. Therefore, by comparing the dynamics of organic osmolytes, antioxidant enzyme activities, and ionic contents in *Thinopyrum ponticum* seedlings under NaCl and Na_2_SO_4_ stresses, this study aims to elucidate the physiological and transcriptional regulatory mechanisms underlying the tall wheatgrass’ response to Na_2_SO_4_. The findings will provide a theoretical basis and practical guidance for the comprehensive utilization of *Thinopyrum ponticum* in saline–alkaline regions.

## 2. Results

### 2.1. Effect of Salt Stress on Growth of Thinopyrum ponticum

**Figure 1:**
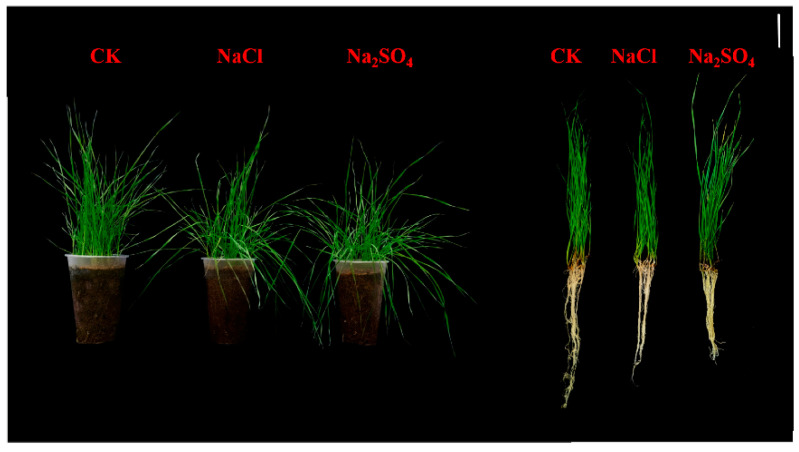
Phenotypes of *Thinopyrum ponticum* in control (CK)-, NaCl (150 mM)-, and Na_2_SO_4_ (150 mM)-treated plants on ninth day of treatment. Scale bars = 6 cm.

Neutral salt stress from both NaCl and Na_2_SO_4_ resulted in phenotypic changes in *Elytrigia elongate* (Figure 1). Compared with CK, NaCl stress resulted in significant reductions in root length and seedling weight but had little effect on leaf length and root weight. Treatment with Na_2_SO_4_, on the other hand, resulted in reductions in root length, seedling weight, and root weight (Table 1).

### 2.2. Physiological Response of Thinopyrum ponticum to Salt Stress

#### 2.2.1. Salt Stress Reduced Relative Water Content of Leaves

**Figure 2:**
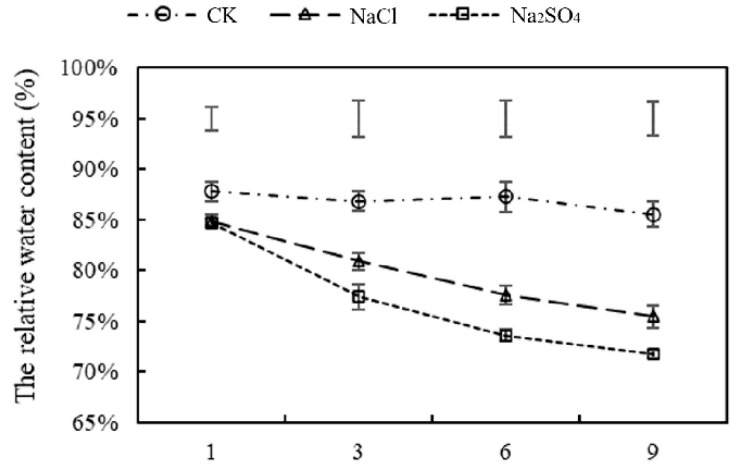
The effects of two kinds of neutral salt stress on the RWC of *Thinopyrum ponticum*. The vertical bars at the top of the figure indicate the least significant difference (LSD) values among treatments on each day of treatment (*p* < 0.05).

Salt stress significantly reduced the relative water content (RWC) in *Thinopyrum ponticum* leaves, with steadily increasing water loss as the duration of salt stress increased. On the first day of salt stress, no significant difference in RWC was detected between NaCl- and Na_2_SO_4_-treated plants. The RWCs of leaves under Na_2_SO_4_ treatment were significantly lower than those of other treatments after three, six, and nine days of salt stress, indicating that water loss in leaves under Na_2_SO_4_ treatment was more severe than under NaCl treatment (Figure 2).

#### 2.2.2. Salt Stress Induced Lipid Peroxidation and Proline Content

**Figure 3:**
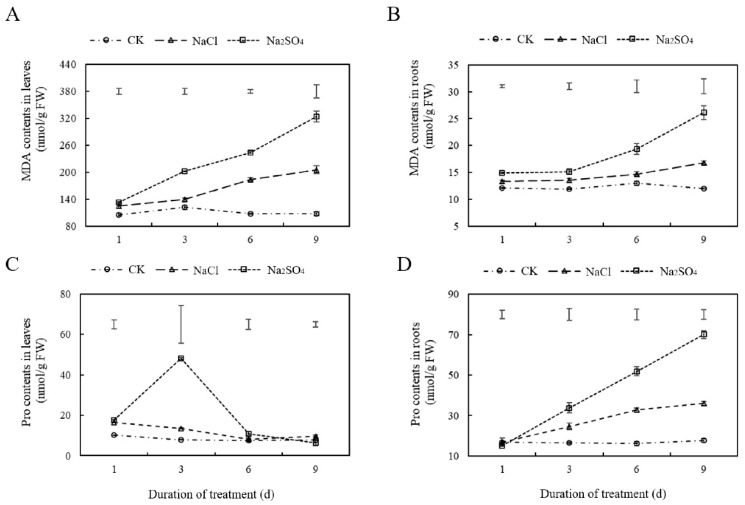
The effects of two kinds of neutral salt stress on MDA (**A**,**B**) and Pro (**C**,**D**) contents in leaves and roots of *Thinopyrum ponticum*. The vertical bars at the top of the figure indicate the least significant difference (LSD) values among treatments on each day of treatment (*p* < 0.05).

The MDA levels in leaves increased significantly under both salt stress treatments, with significant differences between the treatments and the control observed on the third, sixth, and ninth days of treatment (Figure 3A). The MDA levels in roots increased gradually over time under Na_2_SO_4_ stress, while the increase under NaCl stress was more modest, with a significant difference observed only on the ninth day of treatment (Figure 3B). Changes in Pro contents in leaves in response to the two kinds of neutral salt stress were inconsistent. Under NaCl treatment, the levels of Pro were slightly elevated on the first day of stress but subsequently decreased to control levels, while Na_2_SO_4_ treatment produced a sharp increase on the third day of stress (Figure 3C). Changes in Pro content in roots in response to salt treatments were similar to those of MDA (Figure 3D).

#### 2.2.3. Salt Stress Affected POD, SOD, and CAT Activities

**Figure 4:**
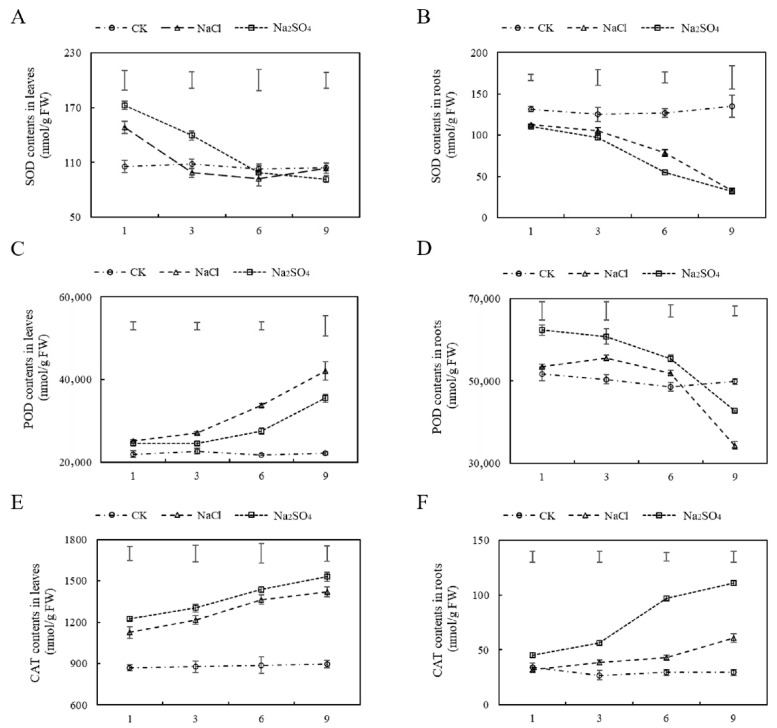
The effects of two kinds of neutral salt stress on SOD (**A**,**B**), POD (**C**,**D**), and CAT (**E**,**F**) activities in leaves and roots of *Thinopyrum ponticum*. The vertical bars at the top of the figure indicate the least significant difference (LSD) values among treatments for each day of treatment (*p* < 0.05).

Compared with CK, the SOD activities in leaves under both types of salt stress were elevated in the early stage of salt stress. Under prolonged salt stress, SOD activities gradually declined and eventually stabilized at levels comparable to CK under NaCl treatment. In contrast, the decline in SOD activity persisted for a longer duration under Na_2_SO_4_ stress, later stabilizing relative to the NaCl group (Figure 4A). The SOD activities in roots decreased gradually under both kinds of neutral salt stress (Figure 4B). The POD and CAT activities in leaves and CAT activities in roots increased steadily with increased treatment time with both salt types. POD activities in leaves under NaCl treatment were higher than those under Na_2_SO_4_ treatment (Figure 4C), while CAT activities in roots were the highest under Na_2_SO_4_ treatment (Figure 4F). No significant differences in CAT activities were observed in leaves between salt treatment (Figure 4E). In addition, the POD activities in roots first increased with salt treatment and then decreased, with significant reductions in POD activities under both types of salt stress observed on the ninth day of treatment relative to the control (Figure 4D).

#### 2.2.4. Salt Stress Affected Ion Content

**Figure 5:**
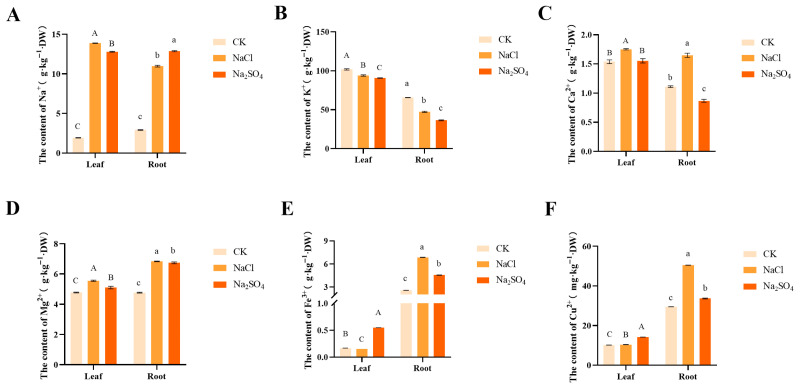
The effects of two kinds of neutral salt stress on Na^2+^ (**A**), K^+^ (**B**), Ca^2+^ (**C**), Mg ^2+^ (**D**), Fe^3+^ (**E**), and Cu^2+^ (**F**) contents in leaves and roots of *Thinopyrum ponticum*. The vertical bars at the top of the figure indicate the least significant difference (LSD) values among treatments for each day of treatment (*p* < 0.05). Capital letters indicate significant differences in leaves and lowercase letters represent significant differences in the roots.

Salt stress markedly elevated Na^+^ concentrations in both leaves and roots. Following NaCl treatment, a preferential accumulation of Na^+^ was observed in the leaves, whereas Na_2_SO_4_ stress led to greater Na^+^ retention in the roots (Figure 5A). Conversely, K^+^ levels were significantly reduced compared to the control group (Figure 5B). Under NaCl stress, Ca^2+^ content exhibited a substantial increase in both leaves and roots; however, Na_2_SO_4_ stress resulted in a pronounced decline in root Ca^2+^ levels (Figure 5C). Root Mg^2+^ uptake was significantly more pronounced than in leaves, with plants subjected to NaCl stress displaying higher Mg^2+^ concentrations than those under Na_2_SO_4_ stress (Figure 5D). Additionally, NaCl stress enhanced the uptake of Fe^3+^ and Cu^2+^ in both leaves and roots of *Thinopyrum ponticum* relative to the control, with root concentrations exceeding those under Na_2_SO_4_ stress (Figure 5E,F).

### 2.3. RNA-Seq Analyses of Leaves and Roots of Thinopyrum ponticum Under Na_2_SO_4_ Stress

#### 2.3.1. Identification of DEGs and Venn Diagram Analysis

Analyses of both morphological phenotypes and physiological indexes indicated that Na_2_SO_4_ stress resulted in a more severe response in *Thinopyrum ponticum*. Therefore, we conducted transcriptomic analyses on *Thinopyrum ponticum* leaves and roots under Na_2_SO_4_ stress treatment using RNA-Seq.

**Figure 6:**
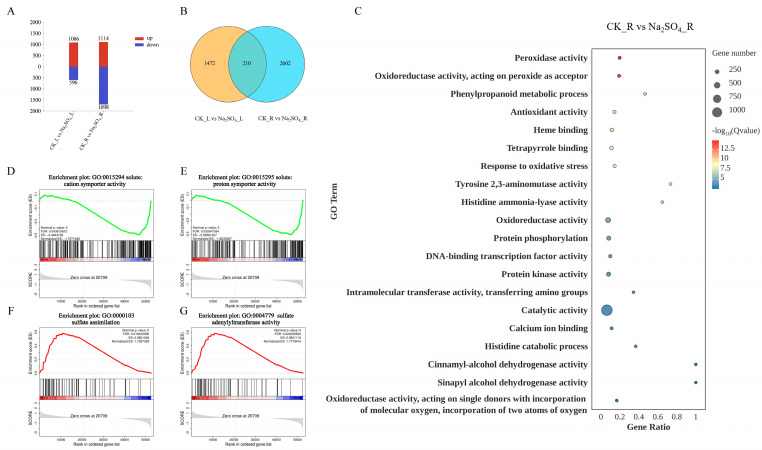
RNA-Seq analyses of *Thinopyrum ponticum* under Na_2_SO_4_ stress. (**A**) The number of DEGs in leaves (CK_L vs. Na_2_SO_4__L) and roots (CK_R vs. Na_2_SO_4__R) under Na_2_SO_4_ stress. (**B**) Venn diagram of DEGs identified in leaves and roots. (**C**) GO enrichment analysis of DEGs of CK_R vs. Na_2_SO_4__R. *X*-axis, Gene Ratio; *Y*-axis, enriched GO terms. (**D**–**G**) Gene Set Enrichment Analysis. The red line signifies pathway activation, with the green line signaling inhibition. (**D**) The pathway of solute: cation symporter activity (GO:0015294). (**E**) The pathway of solute: proton symporter activity (GO:0015295). (**F**) The pathway of sulfate assimilation (GO:0000103). (**G**) The pathway of sulfate adenylyltransferase activity (GO:0004779). *X*-axis, rank in ordered gene list; *Y*-axis, Enrichment Score.

A total of 1682 DEGs were identified between leaves under Na_2_SO_4_ treatment and controls (CK_L vs. Na_2_SO_4__L), with 1086 up-regulated DEGs in the stressed leaves and 596 down-regulated DEGs (Figure 6A). Similarly, 2812 DEGs were identified between Na_2_SO_4_-treated roots and the controls (CK_R vs. Na_2_SO_4__R), of which 1114 were up-regulated and 1698 were down-regulated in the salt-stressed roots. More DEGs were identified in salt-stressed roots than in salt-stressed leaves, with 210 overlapping DEGs identified in both tissues (Figure 6B).

#### 2.3.2. GO Enrichment Analysis

GO enrichment analysis was conducted to characterize the biological functions of the DEGs. The results indicated that several GO terms were significantly enriched, including binding and enzyme activity pathways among root DEGs The results indicated that several GO terms associated with binding and enzyme activity pathways were significantly enriched among the root DEGs (Figure 6C). The binding-related pathways included calcium ion binding, tetrapyrrole binding, and heme binding. The enzyme activity-related GO terms included six related to peroxidase activity, suggesting that redox homeostasis in roots was disrupted after stress. Consistent with this, a large number of genes related to peroxidase activity showed differential expression. Catalytic activity was the most commonly observed GO term among the DEGs, while the GO terms with the highest enrichment ratios were cinnamyl-alcohol dehydrogenase activity and sinapyl alcohol dehydrogenase activity. In addition, DEGs were also enriched for GO terms such as ammonia-lyase activity and transcription regulator activity.

At the same time, the top 20 significantly GO enriched pathways of CK_L vs. Na_2_SO_4__L are visualized in Figure S2. The enrichment pathway is completely different from that in the root system. Many genes were significantly enriched in chromosome-related pathways, microtubules, and the supramolecular fiber pathway. This indicates that Na_2_SO_4_ stress affects the assembly and stability of microtubule skeleton components in leaves, thereby affecting the life activities of plant cells.

#### 2.3.3. Gene Set Enrichment Analysis

Gene Set Enrichment Analysis (GSEA) is able to detect weak but consistent trends among predetermined sets of genes and can complement the GO enrichment analysis of DEGs. Therefore, we performed GSEA on all genes of the CK group and the Na_2_SO_4_ stress group to identify functional changes in *Thinopyrum ponticum* roots in response to salt stress. GSEA identified four important functional categories that were significantly enriched in one of the gene sets that was not previously identified via GO enrichment analysis (Figure 6D–G). Solute: cation symporter activity (GO:0015294) and solute: proton symporter activity (GO:0015295) genes were generally inhibited in salt-stressed roots, while sulfate assimilation (GO:0000103) and sulfate adenylyltransferase activity (GO:0004779) genes were activated, indicating that these categories of genes all played a key role in response to Na_2_SO_4_-induced salt stress.

Genes related to redox homeostasis were identified in both GO enrichment analysis and GSEA. To further understand general trends in redox homeostasis gene expression in roots after stress, we visualized two redox homeostasis GO terms, peroxidase activity (GO:004601) and oxidoreductase activity, acting on peroxide as the acceptor (GO:0016684), using GSEA (Figure S3). The enrichment scores for both functional categories were generally negative, indicating that a large number of genes related to redox homeostasis had reduced expression in salt-stressed roots. Therefore, redox homeostasis was likely disrupted in roots after salt stress.

#### 2.3.4. KEGG Enrichment Pathway Analysis

KEGG pathway enrichment analysis among all the DEGs was performed to reveal several important Na_2_SO_4_-induced pathways (Figure S3). The highest number of DEGs in metabolic pathways is observed in both leaves and roots, and the number of DEGs in roots is more than twice that of leaves, indicating that many biochemical reactions were activated to enhance the adaptation to the salt environment. In addition, 50 DEGs in the root system and 16 DEGs in the leaves are enriched in signal transduction pathways and more DEGs are enriched in environmental adaptation in roots. These results indicate that roots respond faster and more sensitively to salt stress than leaves.

### 2.4. Analysis of Transcriptional Regulation Mechanisms in DEGs Under Salt Stress

DEGs related to transcriptional regulation identified in leaves are shown in Figure S4. A total of 85 TFs, 54 protein degradation genes, 41 protein modification genes, 64 receptor kinases, 36 hormone-related genes, 9 calcium regulation genes (Table S2), 7 G-proteins, and 4 redox genes were identified. DEGs related to transcriptional regulation identified in roots are shown in Figure S5. A total of 185 TFs, 96 protein degradation genes and 109 protein modification genes, 208 receptor kinases, 79 hormone-related genes, 36 calcium regulation genes (Table S2), 5 G-proteins, and 16 redox genes were identified.

#### 2.4.1. TF Analysis of DEGs Related to Transcriptional Regulation

**Figure 7:**
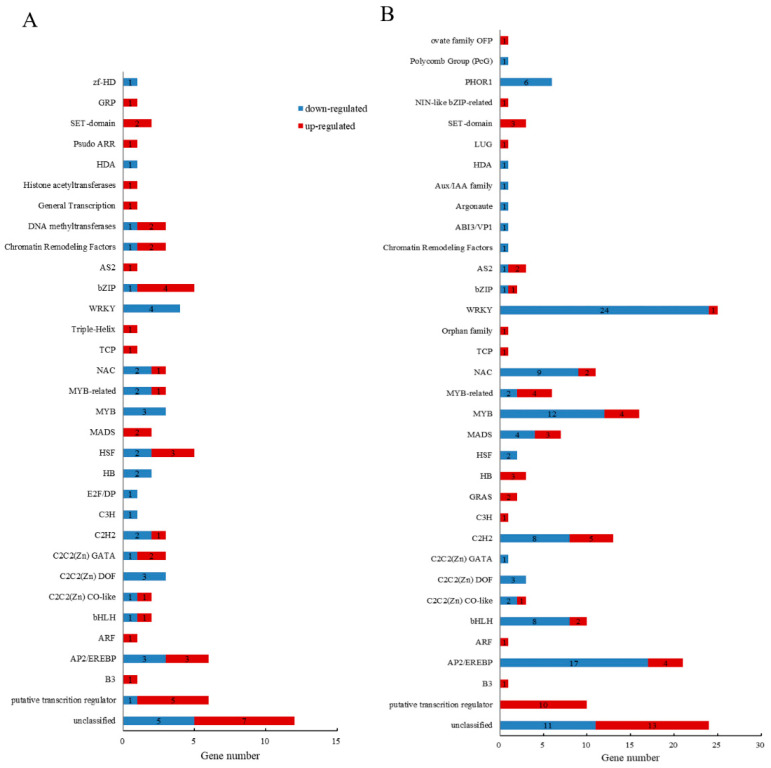
Number of TFs genes among DEGs related to transcriptional regulation. (**A**) DETs annotated in leaves. (**B**) DETs annotated in roots.

Differentially expressed transcription factors (DETs) annotated in leaves and roots were classified and analyzed. A total of 85 DETs were identified in leaves, with 46 up-regulated and 39 down-regulated after Na_2_SO_4_ stress (Figure 7A). The DETs were distributed across 32 transcription factor gene families, among which were many transcription factor families closely associated with plant stress resistance. The most frequently observed families were the AP2/EREBP and HSF families. A total of 185 DETs were identified in the roots, with 69 up-regulated and 116 down-regulated after Na_2_SO_4_ stress (Figure 7B). These DETs were distributed across 34 TF families. Among these, the WRKY transcription factor family was the most frequently observed, with 25 observations (24 down-regulated and 1 up-regulated). The AP2/EREBP TF family was the next most frequently observed family, with 4 up-regulated genes and 17 down-regulated genes. There were 24 common annotated TF families between leaves and roots, with high numbers of DETs in the C2H2, NAC, and MYB families observed in both tissues. It is worth noting that 6 transcripts in leaves (5 up-regulated and 1 down-regulated) and 10 transcripts (all up-regulated) in roots were annotated as putative transcription regulators. These results indicate that *Thinopyrum ponticum* may coordinate many TFs in response to salt stress, though there may be some differences in the responding pathways and genes between leaves and roots.

#### 2.4.2. Analysis of Hormone-Related and Calcium Regulatory DEGs Related to Transcriptional Regulation

**Figure 8:**
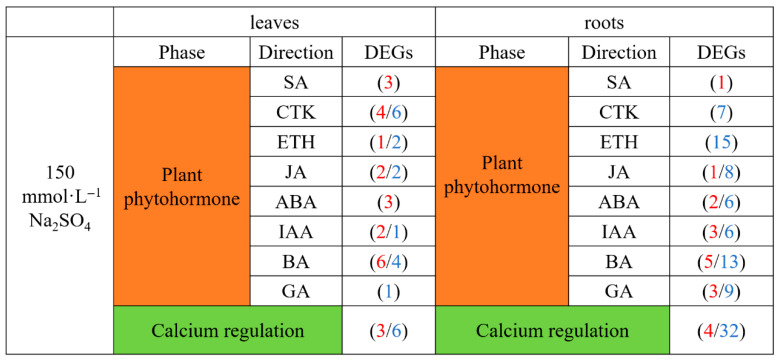
Hormone-related and calcium regulation DEGs involved in salt tolerance in leaves and roots. Red and blue represent up- and down-regulated genes, respectively. Note: IAA: indoleacetic acid; ABA: abscisic acid; BA: brassinosteroid; ETH: ethylene; CTK: cytokinin; JA: Jasmonate; SA: salicylic acid; GA: gibberellin.

Analysis of the genes related to hormone signaling among the DEGs related to transcriptional regulation indicated that the brassinosteroid (BA) pathway was the most enriched hormone pathway among DEGs in both leaves and roots under Na_2_SO_4_ stress (Figure 8). The ethylene (ETH) signal pathway was also enriched among DEGs, with all ETH genes down-regulated after Na_2_SO_4_ treatment. Interestingly, the differentially expressed genes related to salicylic acid (SA) hormones in both leaves and roots were up-regulated. In addition, nine genes related to the calcium signaling pathway were differentially expressed in leaves, with six down-regulated and three up-regulated. Even more calcium signaling pathway genes were differentially expressed in roots, with 4 up-regulated and 32 down-regulated after Na_2_SO_4_ treatment. These results indicated that plant hormone-related genes and calcium signaling pathways may play important roles in the resistance of *Thinopyrum ponticum* to salt stress.

#### 2.4.3. DEGs in SA, ETH Signaling Pathway Signaling, and Prediction of Protein–Protein Interaction (PPI) Networks

##### SA Signaling Pathway

SA is a well-characterized signaling hormone involved in plant defense responses. Under Na_2_SO_4_ stress, although few DEGs related to SA signaling were detected in leaves and roots, all were up-regulated. Further studying these genes may elucidate SA-mediated regulatory mechanisms in *Thinopyrum ponticum* under salt stress.

**Figure 9:**
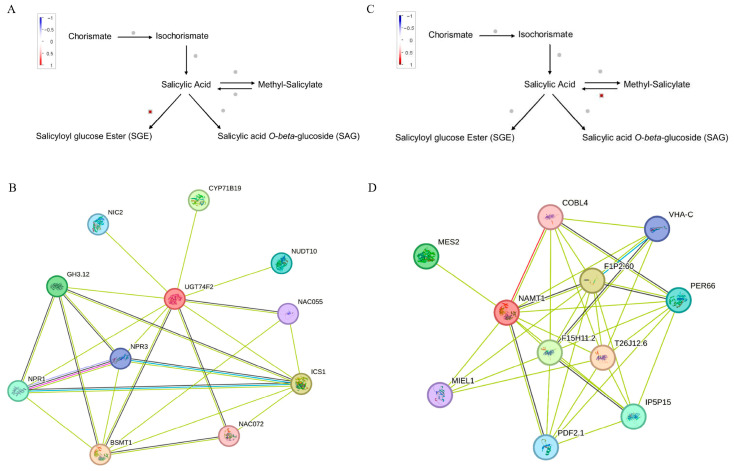
Pathway analysis of differential genes in JA signaling pathway and prediction of protein regulatory network. Regulatory pathways (**A**) and prediction of protein regulatory networks (**B**) of differential genes in JA signaling pathway in leaves. Regulatory pathways (**C**) and prediction of protein regulatory networks (**D**) of differential genes in JA signaling pathway in roots.

In leaves, DEG (*Tel2e01T128800*) encodes *UGT74F2*, a glycosyltransferase that glycosylates benzoic acid derivatives and converts SA to SGE (Figure 9A). Its predicted PPI network indicates interactions with stress-related NAC055 and NAC072 (Figure 9B), as well as with NPR1 and NPR3—substrate adapters of the CUL3–RBX1–BTB E3 ubiquitin ligase complex that mediate target protein ubiquitination and degradation.

In roots, the SA-associated DEG *Tel5e01T317300* encodes *NAMT1*, a methyltransferase crucial for SA biosynthesis (Figure 9C). Predicted PPIs indicate that NAMT1 interacts with PER66, a peroxidase that scavenges H_2_O_2_ and mitigates environmental stresses like wounding, pathogens, and oxidative stress. NAMT1 may also bind MES2 (Methylesterase 2) and play roles in both SA and JA signaling pathways (Figure 9D).

##### ETH Signaling Pathway

**Figure 10:**
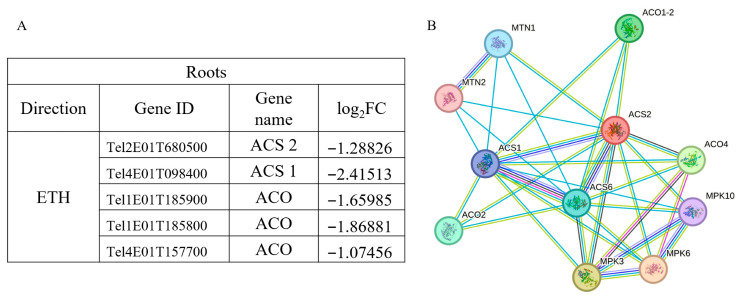
Partial DEGs in root ETH signaling pathway (**A**) and prediction of protein regulatory networks (**B**).

All DEGs associated with the ETH signaling pathway in roots exhibited down-regulation. We further characterized five key DEGs within this pathway, including the direct ethylene precursor gene *ACS* and genes encoding ACC oxidase (*ACO*), both critical for ethylene biosynthesis (Figure 10A). PPI network analysis suggested that ACS proteins may form homomeric interactions and also bind with ACO proteins. Moreover, certain ETH pathway genes are predicted to interact with multiple MPK-related proteins, implying a potential role in oxidative stress-mediated signaling cascades (Figure 10B). The consistent down-regulation of these genes implies that ethylene biosynthesis is likely suppressed in the roots of *Thinopyrum ponticum* during early salt stress.

##### Ca^2+^ Signaling Pathway

**Figure 11:**
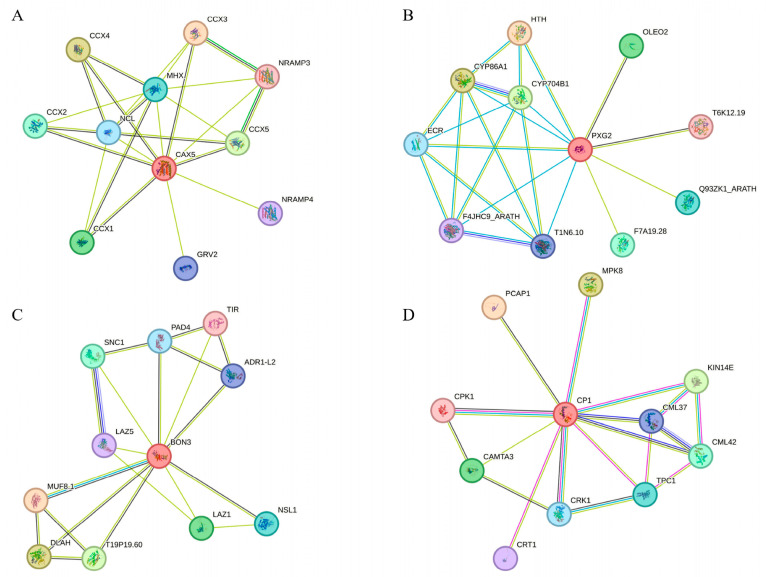
Salt stress-related Ca signaling pathway candidate genes and protein regulatory network prediction. (**A**–**D**) represent the PPI networks of CAX5 (**A**), PXG2 (**B**), BON3 (**C**), and CP1 (**D**), respectively.

Four candidate DEGs associated with Ca^2+^ signaling, namely *PXG2* (*Tel2E01T637200*), *CAX5* (*Tel3E01T773900*), *BON3* (*Tel7E01T696300*), and *CP1* (*Tel4E01T326300*), were selected from both leaves and roots for further analysis based on their fold change in expression levels and functional annotations. PPI networks were predicted for all four candidates, as shown in Figure 11A–D.

As a vacuolar cation/proton exchanger, CAX5 utilizes the proton gradient generated by H^+^-ATPase and H^+^-pyrophosphatase to transport Ca^2+^ and other metal ions into vacuoles. It was predicted to interact with CCX1–5 (Figure 11A), which are H^+^-dependent K^+^/Na^+^ transporters regulating intracellular ion balance. CAX5 may also interact with NCL, which is involved in calcium homeostasis, auxin response, circadian rhythm, flowering timing, and salt stress tolerance. Additional interactors include MHX, NRAMP3, and NRAMP4, which contribute to metal ion distribution and intracellular metal homeostasis.

The PXG2 protein participates in oxygen-dependent signaling pathways and is implicated in plant defense responses. It was predicted to interact with T6K12.19, suggesting a potential role in plant defense mechanisms (Figure 11B).

BON3 is a calcium-dependent phospholipid-binding protein that negatively regulates cell death and defense responses, while also contributing to cellular homeostasis. It was predicted to interact with multiple defense-related proteins, including SNC1, LAZ5, ADR1-L2, PAD4, and TIR (Figure 11C). Additionally, BON3 may interact with NSL1, which negatively regulates SA-mediated programmed cell death during plant immune responses.

CP1 was predicted to interact with multiple Ca^2+^ signaling-related proteins, including CPK1 and CAMTA3, which are involved in signal transduction and plant cold resistance (Figure 11D). It may also interact with TPC1, a voltage-gated inward-rectifying Ca^2+^ channel located on the vacuolar membrane, which also participates in the regulation of stomatal aperture. Additionally, CP1 is predicted to be associated with the MPK8, MKK3–MPK8, and CAMs–MPK8 modules, which negatively regulate ROS accumulation by modulating RBOHD expression during wound response. Interaction with KIN14E, a cytoskeletal hub protein that coordinates microtubules and actin during trichome development, suggests a potential role in the negative regulation of root growth.

### 2.5. Salt-Tolerant Candidate Gene Screening and qRT-PCR Verification

The expression of nine DEGs that were identified as promising candidate genes in response to salt stress were further validated via qRT-PCR (Figure S6). *UGT7472*, *JMT*, and *T4E14.7* were significantly up-regulated and *CAX5* and *PXG2* were significantly down-regulated in the leaves of *Thinopyrum ponticum* under salt stress; *NAMT1*, *BON3*, and *APX7* were significantly up-regulated and *CP1* and *PXG2* were significantly down-regulated in roots. Using qRT-PCR, the relative expressions of antioxidant-related genes, *T4E14.7* and *APX7*, were 146.23 and 560.33 times higher under salt stress, consistent with RNA-Seq results (Figure S7). Thus, these genes may play important roles in the process of salt tolerance in *Thinopyrum ponticum*. The qRT-PCR results showed a strong correlation with the RNA-Seq data, with a coefficient of determination (R^2^) of 0.917, demonstrating the high reliability of the RNA-Seq analyses in this study (Figure S8).

## 3. Discussion

Normally, plants can adjust their metabolic pathways to adapt to changes in the surrounding environment under high salt conditions, including developmental responses such as modulating root architecture, leaf senescence, growth, and biomass allocation [9,31]. The tall wheatgrass *Thinopyrum ponticum* also exhibits such adaptability. The roots are the first tissues that perceive salt stress. Salinity can affect primary and lateral root elongation, inhibit lateral root initiation and organogenesis, and change the direction of root growth [32,33]. Studies have shown that changes in root architecture may be mediated by the redistribution of auxin in roots [34]. Further, salt stress has been found to alter the direction of root growth by reducing the gravity response. In our study, the elongation and growth of the root system were inhibited under salt stress, and the fresh weights of root systems were also reduced. Tarchoune et al. [35] found that the anion-specific effect was only evident under Na_2_SO_4_ treatment, supporting the hypothesis that the stronger reduction in growth caused by Na_2_SO_4_ rather than NaCl was due to the inability of roots to prevent SO_4_ ^2−^ from reaching toxic levels. The changes in root system architecture may be associated with plant salt avoidance.

Stress-induced leaf senescence was previously found to be initiated by a decrease in IAA and an increase in ABA and was promoted by a continuous diminishment in CTKs [36]. Consistent with the results of previous studies, leaf wilting and leaf tip yellowing after treatment with Na_2_SO_4_ were directly observed in this study. Additionally, all DEGs from the CTK signaling pathway in the roots were down-regulated after Na_2_SO_4_ treatment. Our analyses indicated that *Thinopyrum ponticum* may also cooperate with hormone signals, promoting changes in root architecture in response to salt stress. Future work should determine how plants regulate hormones to resolve the conflict between leaf growth and leaf senescence under salt stress.

The activation of osmotic stress pathways, as well as the synthesis and accumulation of compatible osmotic agents, are important strategies for plants to adapt to stress environments [37]. These metabolic responses are common to all osmotic stress pathways and are not specifically induced by excessive salt. In addition, secondary stress induced by salt treatment includes the production of ROS. Low ROS concentration can serve as a signal to activate the salt stress response, while high ROS concentration can damage proteins, lipids, DNA, and carbohydrates [38]. Therefore, the ROS concentration within plant cells must be tightly regulated. Salt stress induces both enzymatic and non-enzymatic systems to alleviate ROS, though these pathways are also activated in response to other stressors in plants. In this study, the MDA contents in leaves and roots significantly increased under salt stress, and Pro accumulated significantly in roots. These results are consistent with those obtained in *Prosopis strombulifera* subjected to both NaCl and Na_2_SO_4_ [39]. At the same time, under the same molar concentration of salt treatment, leaves and roots of *Thinopyrum ponticum* growing under Na_2_SO_4_ treatment showed strong oxidative damage, with increases in both MDA and Pro contents, in relation to the control and NaCl-treated plants. At the same time, the activities of various antioxidant enzymes in the leaves and roots of *Thinopyrum ponticum* were altered under salt stress. The measurement results of antioxidant enzyme activity showed that SOD enzyme activity in the leaves of tall wheatgrass significantly increased after 1 d of salt stress, indicating a rapid response mechanism that enhances the plant’s ability to convert stress-generated free radicals into H_2_O_2_. However, as the stress duration extended, SOD activity gradually declined, leading to a disruption of ROS homeostasis. Additionally, plants continuously produce H_2_O_2_ through multiple pathways under stress conditions, in which CAT acts as a critical second line of defense by scavenging H_2_O_2_. Compared to leaves, the roots, being directly exposed to the stress environment, experienced more severe effects, initially reflected by a marked decrease in SOD activity and subsequent cellular damage. Furthermore, POD, which is often considered a physiological indicator of tissue aging, showed increased activity under salt stress [40]. We speculate that this results from elevated membrane lipid peroxidation, which disrupts reactive oxygen species metabolism, damages membrane structure, and impairs membrane function. As lipid peroxidation products accumulate, plant aging accelerates, thereby activating POD activity. This would indicate that growth inhibition under salt stress was due to lipid peroxidation in tissues and a generalized metabolic disorder.

Excessive Na^+^ can easily undergo transmembrane transport, competitively inhibiting the absorption of K^+^, Ca^2+^, Fe^3+^, and Cu^2+^, disrupting the normal structure of cell membranes, affecting their physiological functions, and causing nutrient deficiencies and deficiencies in the plant body, thereby affecting plant growth and development. This is basically consistent with previous research results [41]. Han et al. [42] demonstrated that plants subjected to salt stress modulate their nutrient uptake and translocation mechanisms to maintain internal nutrient homeostasis, thereby enhancing their adaptability to saline–alkaline environments. Our study revealed that NaCl stress significantly promoted the root absorption of Ca^2+^ and Mg^2+^. Na_2_SO_4_ stress inflicted more severe physiological damage compared to NaCl, leading to the inhibition of Ca^2+^ uptake in roots. Furthermore, Mg^2+^ serves as the primary cellular substitute for potassium ions. Although less efficient than K^+^, Mg^2+^ can partially fulfill K^+^ roles in maintaining charge balance and activating key enzymes. Under conditions where K^+^ uptake is strongly inhibited by Na^+^, tall wheatgrass compensates for potassium functional deficiency by enhancing Mg^2+^ absorption, thereby sustaining essential metabolic processes. This mechanism may represent a crucial adaptive strategy of tall wheatgrass in response to Na_2_SO_4_ stress. Moreover, GO enrichment analysis in roots of Na_2_SO_4_-treated plants revealed that DEGs were significantly enriched for multiple metabolic pathways related to redox homeostasis, demonstrating that salt stress can disrupt the redox homeostasis of plant cells, consistent with previous research findings [43]. In addition, GSEA indicated that a large number of genes involved in redox homeostasis were inhibited in response to early salt stress. Several other metabolic pathways were found to be enriched among DEGs after salt treatment. Cinnamyl alcohol dehydrogenase is known to be a key enzyme in plant secondary metabolism, especially lignin synthesis. Sinapyl alcohol dehydrogenase is also a phenolic compound present in plant cell walls and is involved in lignin synthesis together with other phenolic compounds. These pathways have both been reported to play important roles in plant stress.

Plants can regulate the expression of important genes involved in transcriptional regulation, signal transduction, endogenous hormones, and biosynthesis under salinity stress. With the rapid development of transcriptome sequencing technology, RNA-Seq has been widely used in the study of plant responses to stress [44]. As a complementary approach to GO and GSEA, we identified the putative functions of salt-responsive genes using MapMan [45], which showed salt-induced changes in transcriptional regulation processes. DEGs were categorized into TFs, protein degradation genes, protein modification genes, receptor kinases, hormone-related genes, calcium regulation genes, G-proteins, and redox genes. Increasing evidence suggests that TFs play critical roles as a mechanism of defense under salt stress [46]. In the present study, many TFs were discovered in leaves and roots under salt stress. TFs such as AP2/EREBP, HSF, WRKY, NAC, and MYB were observed to be engaged in the regulation of abiotic stress tolerance in plants. These results suggested that TFs in *Thinopyrum ponticum* play important roles in salt stress response via regulating the transcription of the downstream genes responsible for plant tolerance to salt stress, as has been observed in numerous species of plants.

In addition to other small molecules such as ROS, plant hormones trigger specific signal cascades upon abiotic or biotic stress perception. The fluctuations in several key hormone levels such as ABA, ETH, and JA have been shown to play pivotal roles in response to unfavorable environmental stimuli. Here, we focus on the ethylene signaling pathway and SA signaling pathway. Research on *Cucurbita pepo* has shown that gain-of-function mutations in the ethylene receptors *CpETR1B*, *CpETR1A*, and *CpETR2B* confer enhanced salt tolerance. This improvement is primarily attributed to alterations in cytoplasmic components, characterized by reduced Na^+^ accumulation and increased levels of proline, total carbohydrates, and anthocyanins [46]. Divi et al. [47] showed that either endogenous overproduction of ethylene or treatment with the ethylene precursor ACC can overcome the salt-induced restraint of *Arabidopsis* seed germination. However, the role of ethylene in plant salt tolerance remains elusive. Early investigations have revealed that *Arabidopsis thaliana* exhibits significant induction of *ACS5* and *ACS7* genes under high-salinity conditions [19]. In contrast, a recent study on cotton has yielded divergent findings, demonstrating that salt stress suppresses the expression of several key genes, including *ACS1*, *ACS7*, and *ACO*, within the ethylene biosynthesis and signaling pathway during the early stages of stress [48]. This study is completely consistent with the results obtained from cotton studies, wherein salt stress markedly suppressed the expression of *ACS1*, *ACS2*, and *ACO* genes in roots, demonstrating that ethylene biosynthesis may be inhibited by early salt treatment in *Thinopyrum ponticum*, and this may be a response mechanism of its resistance to salt stress. In addition, our data also found that salt stress significantly up-regulated the gene expression of members of the SA signaling pathway, with significant up-regulation of *NAMT1*, which promotes SA synthesis, in roots. A recent study on *Asarum sieboldii Miq* revealed that transcription factor families including MYB, WRKY, TCP, and bHLH play crucial roles in SA-mediated salt stress responses, with most members exhibiting significant up-regulation [49]. In this study, TCP transcription factors were up-regulated in both leaves and roots under salt stress, which is consistent with previous findings. This suggests that the adaptation of *Thinopyrum ponticum* to salt stress may also be regulated through the interplay between SA and TCP transcription factors. In addition, under salinity stress conditions, SA can regulate transcript levels of *ACS*, *NHX*, *SOS1*, *HKT1*, and *HKT2* to eliminate negative effects of salinity stress-induced ETH production [18,50]. Our data suggests that there may be crosstalk between SA and ETH, which jointly regulate the response to salt stress. In addition, the transcript levels of *UGT74F2* were up-regulated in leaves under salt stress. Although the biological function of SGE is still unclear, predictions of PPI networks suggested that *UGT74F2* may interact with multiple NAC gene family members, indicating that it may also play a role in salt tolerance. Reginato et al. [51] observed that *Prosopis strombulifera* exposed to salinity accumulated high levels of catechol. This phenol is synthesized from the SA pathway by the action of salicylate hydroxylase. These results further proposed that the SA signaling pathway is involved in the plant’s response to alleviate salt stress. Furthermore, ETH can crosstalk with the SA pathway either antagonistically or by promoting them to achieve tailored defense responses; the underlying mechanisms of genes in these two signaling pathways in response to Na_2_SO_4_ stress in tall wheatgrass are still our focus.

High concentrations of Na^+^ and the accumulation of ROS activate the cytoplasmic Ca^2+^ signaling pathway, thereby facilitating the regulation of cellular homeostasis. Several studies have reported that salt-induced Ca^2+^ elevations originate primarily in roots. Recently, the application of advanced Ca^2+^ reporter proteins has yielded critical insights into the calcium signatures elicited by salt stress. Xiong et al. [52] demonstrated that root exposure to NaCl triggers systemic, wave-like Ca^2+^ increases in leaves. In our case, a substantial number of DEGs associated with Ca^2+^ signaling were identified in roots under Na_2_SO_4_ treatment. Most of these genes were down-regulated, indicating that early salt stress may induce a localized Ca^2+^ signal prior to systemic propagation. This observation aligns with findings reported by Choi et al. [53] in *Arabidopsis*. Furthermore, we predicted PPI networks for several DEGs related to Ca^2+^ signaling in both leaves and roots. The encoded proteins were predicted to interact with multiple Ca^2+^-responsive proteins involved in stress responses, including CDPK1 and CAMs-MPK8, indicating their potential role in the salt stress adaptation of *Thinopyrum ponticum*. Among the DEGs, we identified several calmodulin-like (CML) genes, including *CML10* and *CML25/26* (Table S2). CMLs are plant-specific calcium sensors known to participate in signal transduction under salt stress [54]. Notably, the knockout of *AtCML9* in *Arabidopsis* was reported to enhance salt tolerance [55]. We also detected the salt-induced expression of *CDPK* genes, such as *CDPK6* and *CDPK20* (Table S2), which are frequently associated with salt stress responses. Although numerous proteins involved in Ca^2+^ transport and binding contribute to Ca^2+^ signal transduction, the complexity of their interactions means many aspects of the Ca^2+^ signaling network remain unclear. PPI predictions indicated that CP1 may negatively regulate root development through interacting with KIN14E, a finding consistent with our observed root phenotypes.

**Figure 12:**
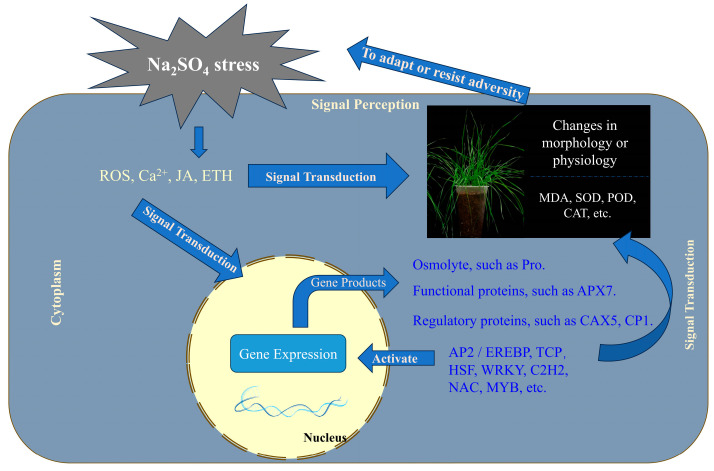
A model diagram of perception, transduction, and response of Na_2_SO_4_ stress signals in *Thinopyrum ponticum*.

Based on the aforementioned research, a conceptual model was formulated to delineate the perception, transduction, and response mechanisms of *Thinopyrum ponticum* to Na_2_SO_4_ stress (Figure 12). Initially, membrane receptors in root cells perceive alterations in external osmotic pressure and Na^+^ concentration, thereby activating Ca^2+^ channels on the plasma membrane. This elicits a rapid influx of Ca^2+^, generating transient Ca^2+^ signals that stimulate a ROS burst and concurrently activate the JA and ETH signaling pathways. These primary signaling molecules act synergistically to amplify downstream responses and initiate a MAPK phosphorylation cascade, thus ensuring efficient propagation of the stress signal. Concurrently, the cell swiftly mobilizes its antioxidant defense apparatus: MDA accumulates as an indicator of membrane lipid peroxidation, while antioxidant enzymes including SOD, POD, and CAT are recruited to collectively mitigate ROS burden. In parallel, osmotic adjustment mechanisms are induced, leading to the accumulation of compatible solutes such as proline to sustain cellular turgor and hydraulic homeostasis. Within the nucleus, an integrated transcriptional regulatory network orchestrated by AP2/EREBP, TCP, HSF, WRKY, C2H2, NAC, and MYB transcription factors is activated. This network up-regulates genes encoding ion regulatory proteins (e.g., CAX5, CP1) and functional proteins such as APX7, establishing a coherent closed-loop system encompassing transcriptional activation, translational output, and feedback control. Ultimately, through the re-establishment of ion homeostasis, enhancement of osmotic equilibrium, and augmentation of antioxidative capacity, long spike wheatgrass attains adaptive resilience to salt stress or initiates programmed defense responses.

This study utilized RNA-Seq to analyze the expression of genes related to transcription factors, calcium signaling, and hormone signaling under salt stress, providing a theoretical foundation for understanding plant salt tolerance mechanisms and offering new insights and directions for marker-assisted selection (MAS) and genomic selection breeding in salt-tolerant crops. Furthermore, as a wild relative of wheat, *Thinopyrum ponticum* holds significant value for distant hybridization and genetic improvement in wheat breeding. This study also provides a theoretical foundation and technical guidance for developing salt-tolerant wheat varieties through targeted gene introgression and molecular breeding strategies. However, relying solely on expression profiling makes it difficult to distinguish core salt-tolerant genes from secondary response factors and fails to reveal post-transcriptional regulatory mechanisms. Future research should integrate multi-omics data, with a focus on hormone signaling and gene interaction networks, and validate key gene functions through gene editing techniques.

## 4. Conclusions

Na_2_SO_4_ treatment had a stronger effect on the growth and physiology of the tall wheatgrass *Thinopyrum ponticum* than treatment with NaCl. GO enrichment analyses and GSEA enrichment of DEGs from Na_2_SO_4_-treated roots indicated that the redox homeostasis-related pathway and ion balance-related pathways played important roles under salt stress. Transcriptional regulation analysis showed that *Thinopyrum ponticum* can coordinate the expression of multiple TF families, hormone signaling pathways, and calcium signaling pathways in response to salt stress to adapt or resist adversity. Moreover, we found that the AP2/EREBP, HSF, WRKY, NAC, and MYB transcription factor families were the most likely candidates to regulate the response to salt stress. Fluctuations in levels of several key hormones such as SA and ETH occurred as early responses to salt stress. In addition, we selected *UGT7472*, *JMT*, *T4E14.7*, *CAX5*, *CP1*, *PXG2*, *NAMT1*, *BON3*, and *APX7* as salt-tolerant candidate genes in *Thinopyrum ponticum*. PPI analysis suggests that their encoded proteins may interact with stress-related proteins. Our study can provide valuable genetic resources for breeding novel salt-tolerant varieties of *Thinopyrum ponticum*, as well as facilitate marker-assisted selection (MAS) and genome-wide association study (GWAS)-based breeding and genetic improvement for salt tolerance in other plant species. The elucidation of the regulatory roles and intricate mechanisms underlying the salinity stress response in *Thinopyrum ponticum*, particularly through functional validation of candidate genes and dissection of SA and ETH hormone signaling cascades, will constitute a major focus of our future research.

## 5. Materials and Methods

### 5.1. Plant Growth Conditions and Treatments

The seeds of the tall wheatgrass *Thinopyrum ponticum* cv. “Orbit” were purchased from Beijing Baist Grass Industry Co., Ltd. (Beijing, China). Cleaned seeds were planted in a seedling pot (diameter 6 cm × height 14 cm). Seedlings were cultured using the sand culture method (The sand substrate used in the experiment is river sand with a particle size of about 1–3 mm. Before use, rinse it with distilled water 2–3 times and dry it in an oven at 80 °C before conducting the experiment.) and placed in the greenhouse of the Chinese Academy of Forestry, with a 16 h/8 h photoperiod at 25 °C/20 °C (day/night) and 50% relative humidity. After 95% of the seeds germinated, Hoagland nutrient solution was applied, with nutrient solution replenished every three days. After 25 days of continuous culture, when plants were at the 3–4 leaf stage, plants were treated with either 150 mM NaCl or 150 mM Na_2_SO_4_ or left untreated. The untreated control group (CK) received only Hoagland nutrient solution. The leaves and roots of different treatments were collected at 1 d, 3 d, 6 d, and 9 d after the start of treatment. Collected tissues were frozen rapidly in liquid nitrogen and stored in a −80 °C ultra-low temperature freezer for subsequent physiological and transcriptome analyses. Three replicates were collected for each treatment.

### 5.2. Determination of Growth and Physiological Indexes

Fifteen seedlings of *Thinopyrum ponticum* were collected on the 9th day of treatment for the CK, NaCl, and Na_2_SO_4_ treatment groups. The seedling lengths, root lengths, seedling weights, and root weights were measured, with three replicates for each treatment.

The physiological indexes of leaves and roots from different treatments at different time points were measured, including malondialdehyde (MDA) and proline (Pro) levels, as well as catalase (CAT), peroxidase (POD), and superoxide dismutase (SOD) activities. The kits to measure these were purchased from Beijing Solarbio Technology Co., Ltd. and used according to the manufacturer’s instructions.

After collection, the fresh weights (FW) of the leaves of seedlings from different treatments groups were quickly measured. The leaves were soaked in deionized water for 24 h while being kept in shade; after removal, moisture on the surfaces of the leaf blades was dried with absorbent paper, and the saturated weights (TW) of leaf blades were quickly measured. The leaves were then placed in an oven at 105 °C for 30 min and dried to constant weight at 80 °C. The dry weights (DW) were then measured, and the relative water content (RWC) of each sample was calculated using the following formula: RWC (%) = [(FW − DW)/(TW − DW)] × 100% [56].

### 5.3. Determination of Ions

Leaves and roots from different treatment groups were collected separately. They were initially withered in a 105 °C oven for 30 min and then dried at 70 °C until constant weight was achieved. The dried samples were ground using a small grinder (BF-08, Hebei Benchen Technology Co., Ltd., Shijiazhuang, China), passed through a 0.28 mm sieve, and each sample was processed in triplicate. A total of 0.5 g of each sample (accurately weighed to 0.0001 g) was digested with a mixture of nitric and perchloric acids. Cations (Na^+^, K^+^, Ca^2+^, Mg^2+^, Fe^3+^, and Cu^2+^) were measured using an atomic absorption spectrophotometer (GGX-600, Beijing Haiguang Instrument Co., Ltd., Beijing, China) [35]. Ion content analysis was conducted by Wuhan ProNets Testing Technology Co., Ltd. (Wuhan, China).

### 5.4. RNA Extraction and Transcriptome Sequencing

The RNA Easy Fast Plant Tissue Kit for RNA rapid extraction (Tiangen #DP452) was used to extract RNA from the leaves and roots of CK and Na_2_SO_4_-treated plants after 1 day of treatment. Each treatment comprised 3 biological replicates, yielding a total of twelve samples. The integrity of RNA and the presence of DNA contamination were detected via 1% agarose gel electrophoresis. A NanoPhotometer spectrophotometer was used to detect RNA purity, and an Agilent 2100 bioanalyzer (Shanghai Biotechnology Co., Ltd., Shanghai, China) was used to accurately determine RNA integrity (Figure S1).

High quality RNA was used to construct high-throughput RNA sequencing libraries. RNA sequencing was performed by Guangzhou Jidiao Biotechnology Co., Ltd. (Guangzhou, China). Quality control on raw reads was assessed using fastp software (fastp 0.18.0) [57], and low-quality data were filtered to obtain clean reads. Clean reads were then aligned to the *Thinopyrum elongatum* (diploid) reference genome by using HISAT2 v2.1.0 [58]. Gene expression levels were normalized using the TPM (Transcripts Per Million) method to account for variations in gene length and sequencing depth, enabling comparative analysis across samples and genes. Differential expression analysis between the two groups was performed using the DESeq Bioconductor Package. Differential expression was determined based on the negative binomial distribution model. Benjamini and Hochberg correction was used to control the false discovery rate. Differentially expressed genes (DEGs) were identified using thresholds of |log_2_(fold change)| > 1 and a false discovery rate (FDR) < 0.05 to adjust for multiple comparisons [59].

### 5.5. Pathway Enrichment Analysis

The Gene Ontology (GO) enrichment analysis of the functionally annotated DEGs was implemented by the topGO (2.46.0 version, corrected *p*-value < 0.05 based) Wallenius non-central hyper-geometric distribution [60]. Gene Set Enrichment Analysis (GSEA) enrichment was performed to evaluate whether the predefined gene sets showed a significant up- or down-regulation between the experimental group and the control group relative to the set of all genes. Pathways with |Normalized Enrichment Scores (NES)| > 1, *p*-value < 0.05, and FDR q-value < 0.25 were classified as significantly enriched. Furthermore, to analyze the *Thinopyrum ponticum* transcriptome, all of the unigenes were submitted to the Kyoto Encyclopedia of Genes and Genomes (KEGG) pathway database for the systematic analysis of gene functions. KOBAS software (v2.0.12 version, corrected *p*-value < 0.05) was used to test the statistical enrichment of DEGs in KEGG pathways.

### 5.6. Prediction and Classification of Transcriptional Regulatory Factors

The genome annotation file of *Thinopyrum ponticum* was obtained from Mercator version 3.6 (https://www.plabipd.de/mercator_main.html). The gene IDs and log_2_ fold change expression values of DEGs from leaves and roots of *Thinopyrum ponticum* were input into MapMan 3.5.1R2, and the functions and expression of DEGs related to transcriptional regulation were then graphically displayed [61].

### 5.7. Protein–Protein Interaction (PPI) Network Analysis

The online website String was used to illustrate the protein–protein interaction (PPI) network diagrams (https://cn.string-db.org/cgi/input?sessionId=bsVNrd9LngSC&input_page_active_form=multiple_identifiers).

### 5.8. Quantitative Reverse Transcription Polymerase Chain Reaction (qRT-PCR) Confirmation

Nine candidate genes related to salt tolerance were selected from leaves and roots, and qRT-PCR analysis was performed to verify the results of high-throughput sequencing. *Actin7* (*ACT*) and *Glyceraldehyde-3-phosphate dehydrogenase* (*GADPH*) were used as reference genes. The 2^−ΔΔCT^ method was used to calculate the relative gene expression values [57]. The gene-specific primers were designed using the online tool Primer-BLAST (Primer designing tool (nih.gov)) and the primers’ sequences are listed in Table S1.

qRT-PCR reactions with total volumes of 10 μL were mixed, containing 2.5 μL of diluted template (10 μL cDNA diluted in 90 μL of ddH_2_O), 1.7 μL of ddH_2_O, 0.8 μL each of upstream and downstream primers (10 μM), and 5 μL of 2 × M5 HiPer SYBR Premix Es Taq (with Tli RNaseH) (Mei5 Biotechnology Co.,Ltd., Beijing, China). Reactions were then run on a LightCycler480 System (Roche, Shanghai, China) with initial denaturation of 95 °C for 30 s (Ramp rate: 4.4 °C/s), 40 cycles of 95 °C for 5 s (Ramp rate: 4.4 °C/s), and 60 °C for 30 s (Ramp rate: 2.2 °C/s; Acquisition Mode: Single), and then 95 °C for 5 s (Ramp rate: 4.4 °C/s), 60 °C for 60 s (Ramp rate: 2.2 °C/s), 95 °C for 1 s (Ramp rate: 0.11 °C/s, Acquisition Mode: Continuous; Acquisitions: 5 per °C), and 50 °C for 30 s (Ramp rate: 2.2 °C/s). Three biological replicates and two technical replicates were performed.

### 5.9. Data Analysis

Growth and physiological data from the three treatment groups were analyzed using analysis of variance (ANOVA). Significant differences between means were determined using Fisher’s protected least significant difference (LSD) test at a significance level of 0.05, performed with SPSS version 19.0 for Windows (SPSS Inc., Chicago, IL, USA).

## Figures and Tables

**Table 1:** Effects of two kinds of neutral salt stress on growth index of *Thinopyrum ponticum*.

Treatment	Mean Seedling Length (cm)	Mean Root Length (cm)	Mean Seedling Weight (g)	Mean Root Weight (g)
CK	25.64 ± 0.62 a	18.82 ± 0.70 a	2.29 ± 0.10 a	1.10 ± 0.01 a
NaCl	23.94 ± 0.60 a	16.16 ± 0.27 b	2.09 ± 0.15 b	1.07 ± 0.03 a
Na_2_SO_4_	26.08 ± 0.94 a	14.50 ± 0.45 b	1.64 ± 0.07 c	0.96 ± 0.05 b

Note: Different lowercase letters represent significant differences (*p* < 0.05). Value after “±” represents standard error.

## Data Availability

All data generated or analyzed in this study are included in this article and the Supplemental files. The raw RNA sequencing data were submitted to the NCBI database with the BioProject ID: PRJNA1123323.

## Supplementary Materials

The following supporting information can be downloaded at: https://www.mdpi.com/article/10.3390/plants14172771/s1, Figure S1: Total RNA extracted from the leaves and roots of *Elytrigia elongata.* DL2000 Marker was used. The samples from left to right are: CK-L (three replicates, the control of leaves), CK-R (three replicates, the control of roots), Na_2_SO_4_-L (three replicates, the Na_2_SO_4_ treatment of leaves), and Na_2_SO_4_-R (three replicates, the Na_2_SO_4_ treatment of roots); Figure S2: GO enrichment analysis of DEGs of CK_L vs Na_2_SO_4__L. X-axis, Gene Ratio; Y-axis, enriched GO terms; Figure S3: GSEA analysis of pathways related to redox homeostasis. X-axis, Rank in ordered gene list; Y-axis, Enrichment Score; Figure S4: KEGG enrichment analysis of DEGs between leaves (A) and roots. X-axis, Gene number; Y-axis, Pathway; Figure S5: Overview of DEGs related to transcriptional regulation in leaves under Na_2_SO_4_ treatment. The color scale displays the log_2_fold change values; Figure S6: Overview of DEGs related to transcriptional regulation in roots under Na_2_SO_4_ treatment. The color scale displays the log_2_fold change values; Figure S7: Results of the qRT-PCR analysis; Figure S8: qRT-PCR expression analysis of salt tolerance related candidate genes; Table S1. Candidate salt tolerance genes and corresponding qRT-PCR primers; Table S2. DEGs in Ca^2+^ signaling pathway visualized using MapMan.

## Abbreviations

ABA: Abscisic acid; ACC: 1-aminocyclo-propane-1-carboxylic acid; ACS: ACC synthase; CAT: catalase; DEGs: differentially expressed genes; ETH: ethylene; MDA: malondialdehyde; Pro: proline; POD: peroxidase; ROS: reactive oxygen species; RWC: relative water content; SA: salicylic acid; SOD: superoxide dismutase; SOS: salt overly sensitive; TF: transcription factor.
