# Supplementary material for: Physiological and Transcriptional Regulation of Salt Tolerance in Thinopyrum ponticum and Screening of Salt-Tolerant Candidate Genes
Source: Plants (Basel). 2025 Sep 4;14(17):2771. doi: 10.3390/plants14172771 (PMC12430373; doi:10.3390/plants14172771)
Supplement: Supplementary file 1 [file plants-14-02771-s001.zip › Table. S1.pdf]

Table. S1 Candidate salt tolerance genes and corresponding qRT-PCR primers

Gene ID	Gene name	Gene name abbreviation or Identifier	The tissue sources of DEG	Forward primer (5’ - 3’)	Reverse primer (5’ - 3’)	Product length
Tel2E01T128800	*Uridine diphosphate glycosyltransferase 74F2*	*UGT74F2*	leave	TGGCAAACCTTTTCTTTGGG	ACCACTAACAATTGCCTCCA	193
Mstrg.26995	*Jasmonic acid carboxyl methyltransferase*	*JMT*	leave	CTCCGTAAAATGAACGAGCC	CACCGGGATTTGGTTTATCG	162
Tel3e01T773900	*Calcium-transporting ATPase 5*	*CAX5*	leave	ACAGCAGCATGTAGGGATTT	TTTCTTGCGAGGGTATGTGT	133
Tel1E01T058700	*1-aminocyclopropane-1-carboxylate oxidase*	*T4E14.7*	leave	AGGGACTACGTCAAGCAGTA	ATAGCCGGAAAACGGAGAG	70
Tel2E01T637200	*peroxygenase-like*	*PXG2*	leave, root	TTTACGTGCACAACATCCAC	TTCTCGAAGTTCACTGGCAT	91
Tel5E01T317300	*Inactive anthranilate O-methyltransferase 1*	*NAMT1*	root	GTATGGACCCTCTGGTGAAG	GCTCGACTATTATCGGCTCA	170
Tel7E01T696300	*BONZAI 3*	*BON3*	root	ATTGTGAGGGCATCAGAGTT	TTCTTCATGCGAGCAAACTG	164
Tel4E01T326300	*Calcium-binding protein CP1*	*CP1*	root	GATCCGCGCCATGATAAGTA	TCAGACGATGGCATCAAAGT	100
Mstrg.54633	*L-ascorbate peroxidase 7*	*APX7*	root	CCACTTCAGGCTGGTATTCT	AGGCCTAGACCTTCCAAGTA	
Tel5E01T799600	*Actin 7*	*ACT*	-	CATACTGTGCCGATCTACGA	ATAGTCAAGGGCAACGTAGG	189
Tel7E01T538500	*Glyceraldehyde-3-phosphate dehydrogenase*	*GADPH*	-	ACTGTGGATGTGTCAGTTGT	AAAGTCGGTGGAGACCAAAT	150
