# Supplementary material for: Physiological and Transcriptional Regulation of Salt Tolerance in Thinopyrum ponticum and Screening of Salt-Tolerant Candidate Genes
Source: Plants (Basel). 2025 Sep 4;14(17):2771. doi: 10.3390/plants14172771 (PMC12430373; doi:10.3390/plants14172771)
Supplement: Supplementary file 1 [file plants-14-02771-s001.zip › Table. S2.pdf]

Table. S2 DEGs in Ca^{2+} signaling pathway visualized using MapMan

Roots			Leaves		
Gene ID	Gene name	Log_2FC	Gene ID	Gene name	Log_2FC
Tel1E01T571900	Ca^{2+} -ATPase 7	-1.40983	Tel1E01T225500	GELPs	7.2159376
Tel1E01T573300	Ca^{2+} -ATPase 7	-1.15078	Tel2E01T413000	CFC21_023047	2.1858666
Tel1E01T396000	CML 10	-1.14262	Tel2E01T637200	peroxygenase-like	-7.3721395
Tel2E01T380800	CDPK 20	-1.22409	Tel2E01T606900	CML 22	-1.062699
Tel2E01T308600	IQM 2	-1.85487	Tel3E01T773900	Ca^{2+} -ATPase 5	-8.257388
Tel2E01T637200	peroxygenase-like	-8.29309	Tel3E01T783700	CML 10	-1.105533
Tel2E01T638100	peroxygenase-like	2.868793	Tel3E01T785800	CML 10	2.432444
Tel3E01T358200	IQM 1	-2.17267	Tel3E01T080900	CML 16	-1.1215401
Tel3E01T514600	PBP 1	-1.96955	Tel5E01T123900	CALML 5	-1.1550237
Tel3E01T805700	CML 31	-9.22159			
Tel3E01T783700	CML 10	-1.37974			
Tel3E01T081800	CBP 60	-1.35706			
Tel3E01T785800	CML 10	-1.29944			
Tel3E01T080900	CML 16	-1.18571			
Tel4E01T211600	CML 25/26	-2.12822			
Tel4E01T542600	IQM 14	-2.6409			
Tel4E01T387400	CLB	-1.55227			
Tel4E01T326300	CP1	-1.47302			
Tel5E01T420200	EFCaBP	-2.56651			
Tel5E01T265100	CML 25/26	-1.12701			
Tel5E01T420500	EFCaBP	-2.22147			
Tel5E01T419800	EFCaBP	-1.89419			
Tel5E01T152500	FLO 11	-1.11691			
Tel5E01T124000	CALML 5	-9.76321			
Tel5E01T123900	CALML 5	-1.52143			
Tel5E01T419600	EFCaBP	-3.2942			
Tel5E01T419900	EFCaBP	-3.28911			
Tel5E01T420400	EFCaBP	-2.85167			
Tel6E01T786400	CDPK 6	-1.81848			
Tel7E01T896900	CML 25/26	-3.53838			
Tel7E01T759700	PBP 1	-1.05678			
Tel7E01T250900	CBP	-1.00176			
Tel7E01T466300	CDPK 21	-2.21539			
Tel7E01T696300	BONZAI 3	7.235217			
Tel7E01T753100	Syt 3	1.088714			
Mstrg.46277	Calreticulin	1.57899			
