# Supplementary figures and images for: Physiological and Transcriptional Regulation of Salt Tolerance in Thinopyrum ponticum and Screening of Salt-Tolerant Candidate Genes
Source: Plants (Basel). 2025 Sep 4;14(17):2771. doi: 10.3390/plants14172771 (PMC12430373; doi:10.3390/plants14172771)

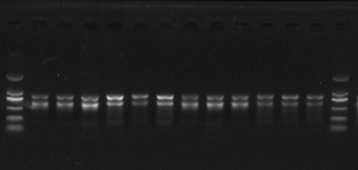

Supplement: Supplementary file 1 [file plants-14-02771-s001.zip › Figure S1.jpg]

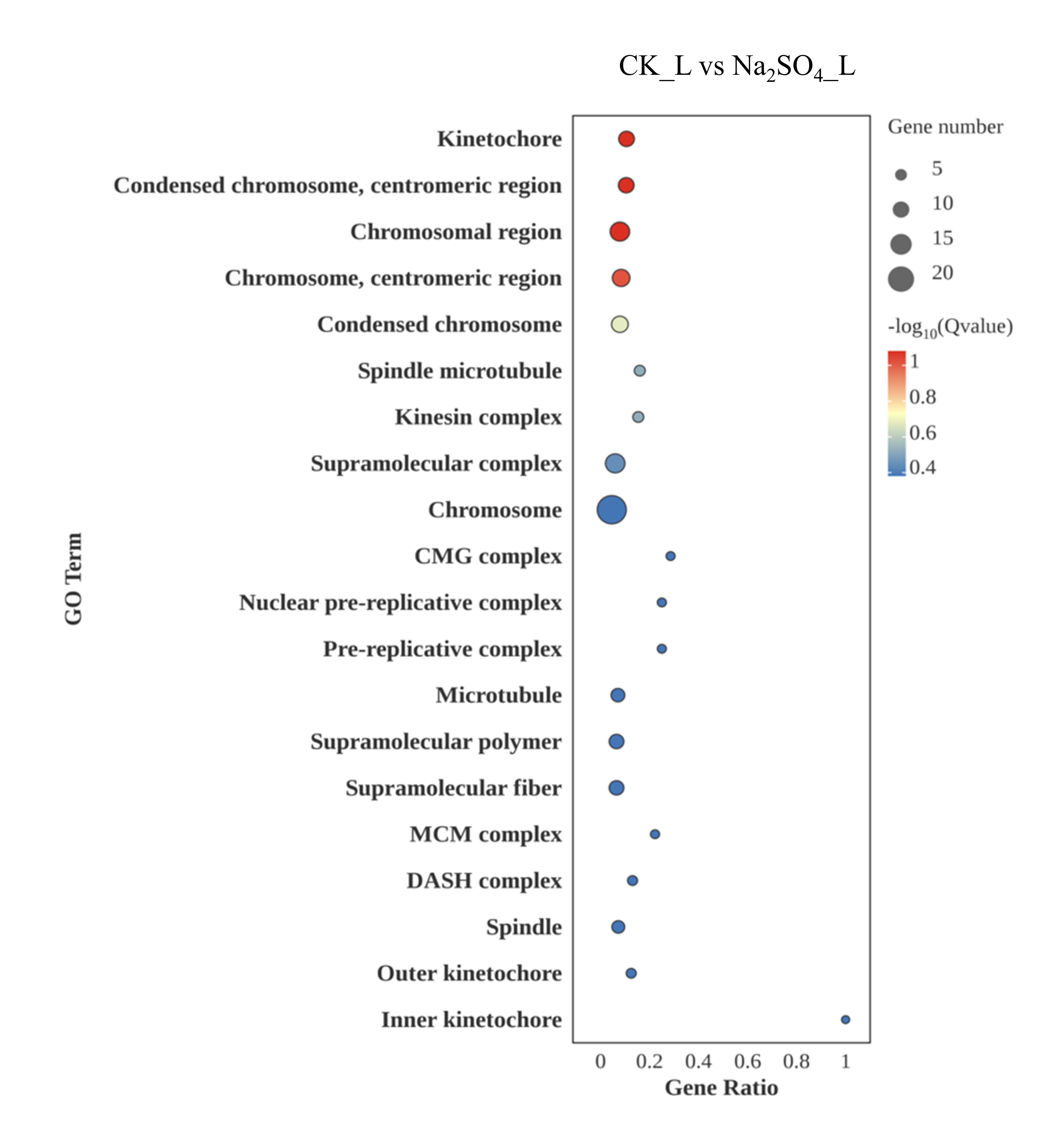

Supplement: Supplementary file 1 [file plants-14-02771-s001.zip › Figure S2.jpg]

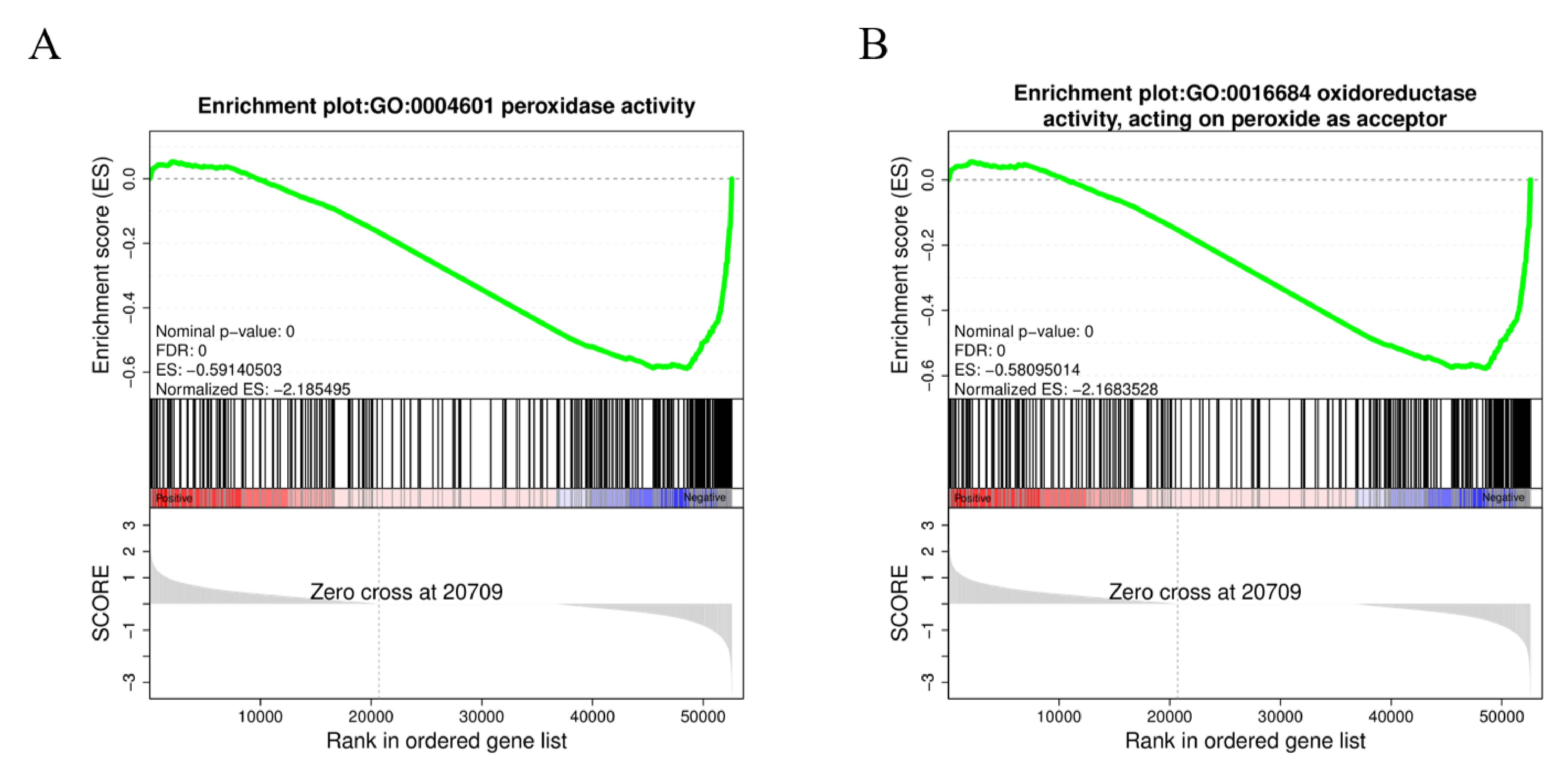

Supplement: Supplementary file 1 [file plants-14-02771-s001.zip › Figure S4.jpg]

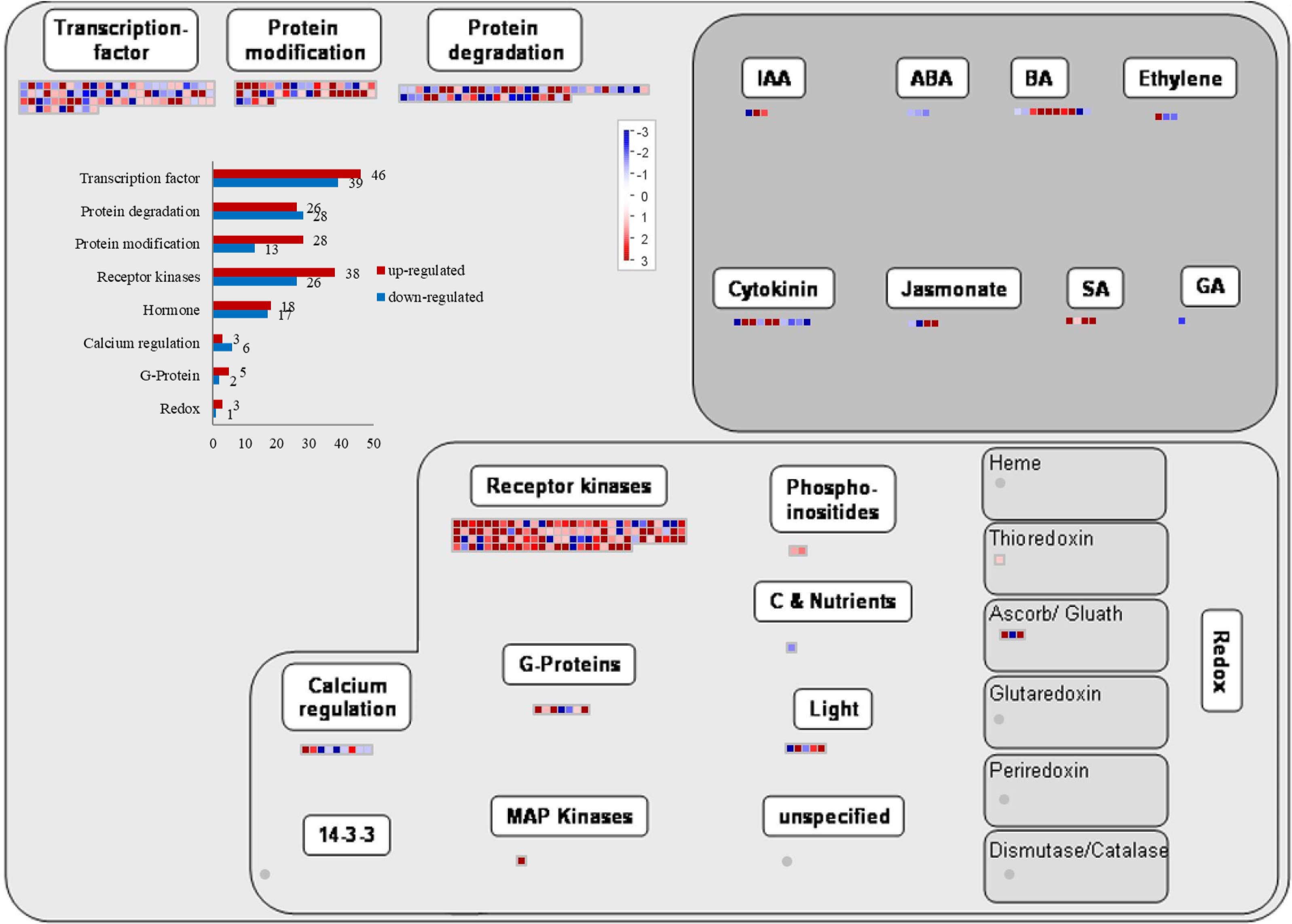

Supplement: Supplementary file 1 [file plants-14-02771-s001.zip › Figure S5.jpg]

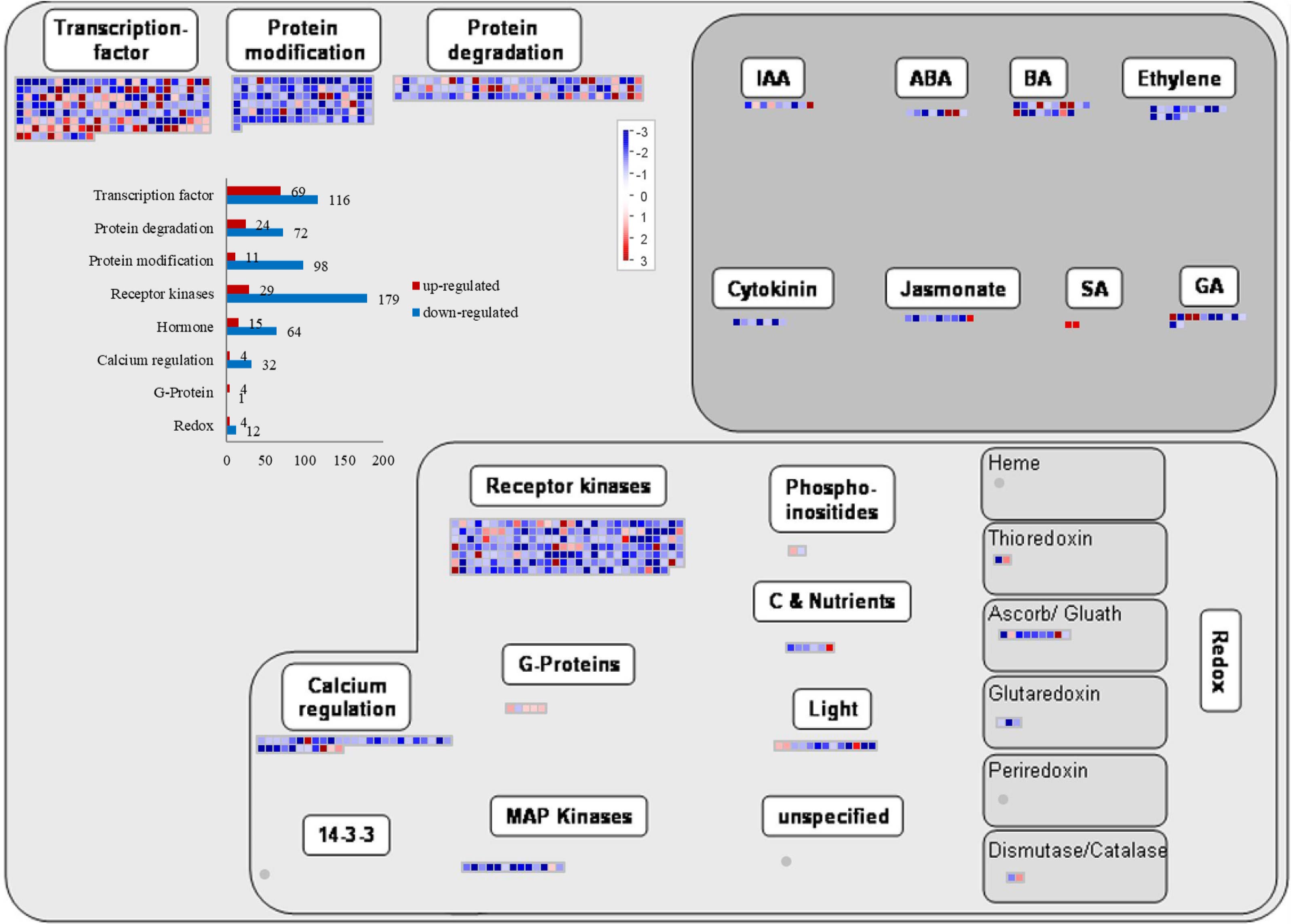

Supplement: Supplementary file 1 [file plants-14-02771-s001.zip › Figure S6.jpg]

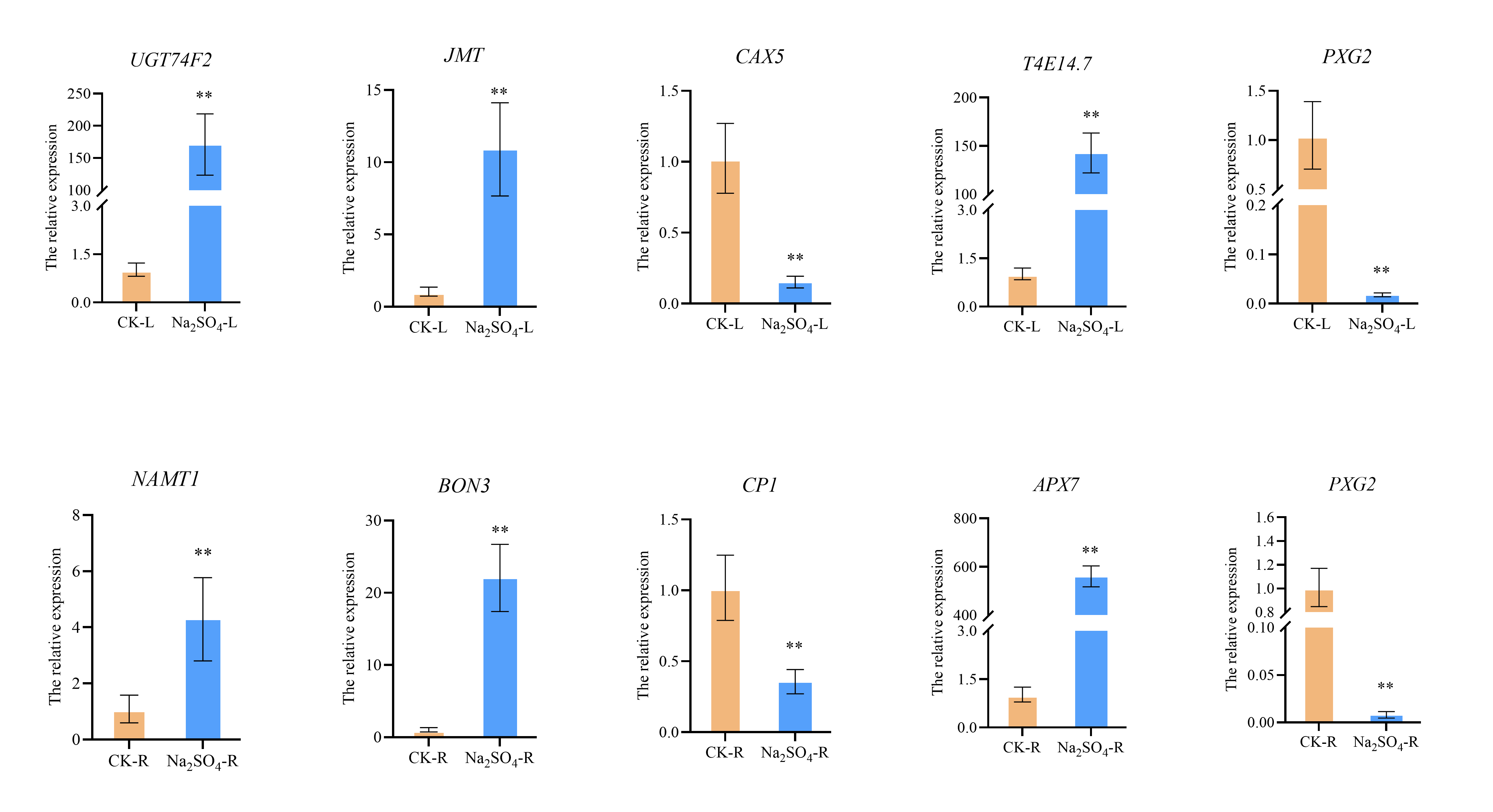

Supplement: Supplementary file 1 [file plants-14-02771-s001.zip › Figure S7.tif]

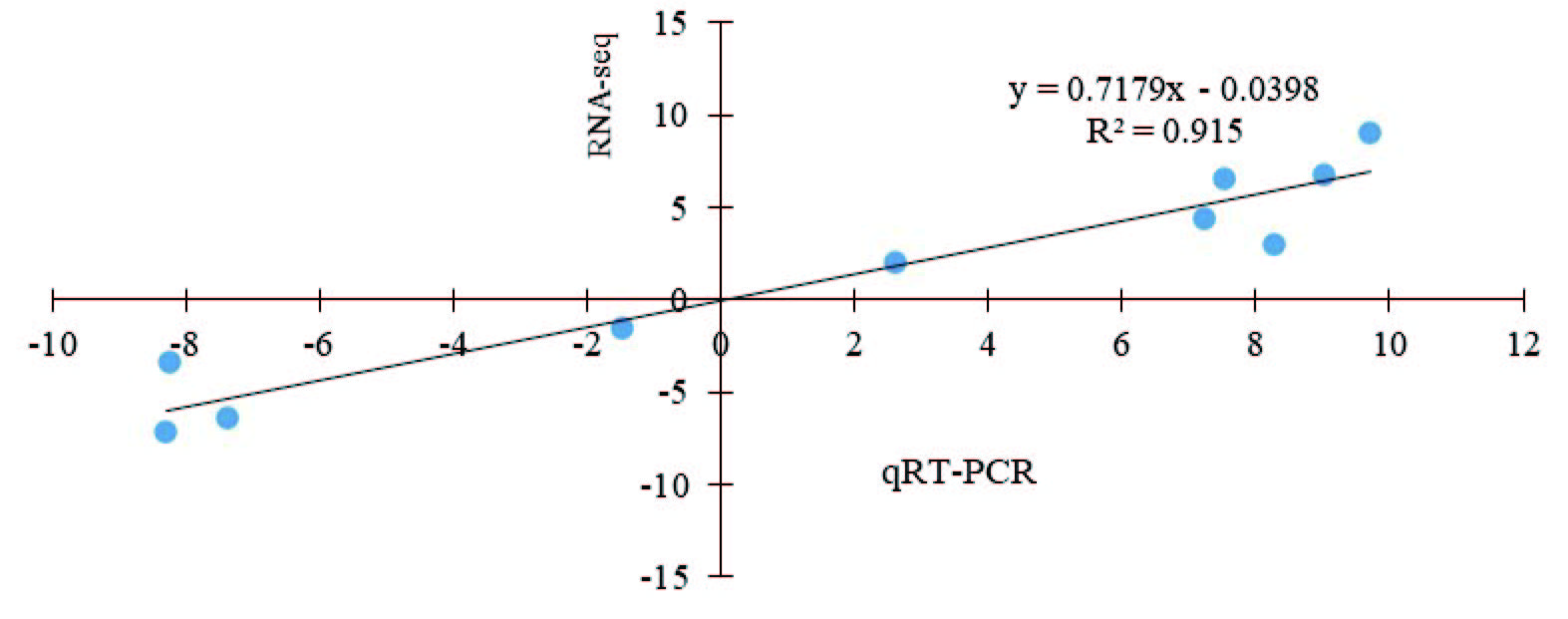

Supplement: Supplementary file 1 [file plants-14-02771-s001.zip › Figure S8.jpg]
